# A fast machine learning dataloader for epigenetic tracks from BigWig files

**DOI:** 10.1093/bioinformatics/btad767

**Published:** 2024-01-04

**Authors:** Joren Sebastian Retel, Andreas Poehlmann, Josh Chiou, Andreas Steffen, Djork-Arné Clevert

**Affiliations:** Machine Learning Research, Pfizer Worldwide Research Development and Medical, Friedrichstraße 110, Berlin 10117, Germany; Machine Learning Research, Pfizer Worldwide Research Development and Medical, Friedrichstraße 110, Berlin 10117, Germany; Machine Learning Research, Pfizer Worldwide Research Development and Medical, Friedrichstraße 110, Berlin 10117, Germany; Machine Learning Research, Pfizer Worldwide Research Development and Medical, Friedrichstraße 110, Berlin 10117, Germany; Machine Learning Research, Pfizer Worldwide Research Development and Medical, Friedrichstraße 110, Berlin 10117, Germany

## Abstract

**Summary:**

We created *bigwig-loader*, a data-loader for epigenetic profiles from BigWig files that decompresses and processes information for multiple intervals from multiple BigWig files in parallel. This is an access pattern needed to create training batches for typical machine learning models on epigenetics data. Using a new codec, the decompression can be done on a graphical processing unit (GPU) making it fast enough to create the training batches during training, mitigating the need for saving preprocessed training examples to disk.

**Availability and implementation:**

The *bigwig-loader* installation instructions and source code can be accessed at https://github.com/pfizer-opensource/bigwig-loader

## 1 Introduction

During the last few years, training machine learning models that take a fixed length sequence found in a genomic region as an input and predict the presence of epigenetic marks measured in experiments like DNAse-seq, Chip-seq, and ATAC-seq has become popular (Zhou and Troyanskaya 2015, Kelley *et al.* 2016, 2018, Avsec *et al.* 2021, Novakovsky *et al.* 2023). These models mostly predict epigenetic marks found in multiple experiments at the same time in a multitask fashion. The individual tasks can either be the binary prediction of the presence of epigenetic marks in a central window, determined by a peak caller, or the regression of the actual epigenetic profiles along the sequence dimension. To directly compare these two approaches, one can apply a threshold to the predictions of the regression task, thereby converting it to classification. Doing so, it has been shown that regression of the measured profiles leads to higher performance (Toneyan *et al.* 2022).

In these two settings, the neural networks can have similar architecture, only the last few layers determining the output dimensionality must be changed. However, the data loading process, providing the model with training examples, must be changed quite a bit more. The reason for this is that loading data from .bed files, in which peaks called by a peak caller are often stored, is fast. Therefore, it is easy to write a data loader doing this on the fly while the model is being trained. When training to predict whole profiles, more data need to be ingested for each training step. The most widely used format for this type of data is the BigWig format (Kent *et al.* 2010). The format stores profiles along the genome in compressed blocks. There exists a relatively fast python library called p*yBigWig* that given a genomic region, determines which blocks to pull from disk, decompresses those blocks and converts the decompressed rows of intervals with their corresponding values into a series of values representing the epigenetic profile (Ryan *et al.* 2023).

The downside of this library is though that it does so for one BigWig file at a time and one region at a time. For model training, each training batch consists of data from many regions in many BigWig files. This makes *pyBigWig* too slow to keep the GPU, used for the training of the neural network, saturated. Dataloading becomes the bottleneck even when training a very large model. To prevent this from happening, researchers preprocess data from BigWig files, creating the training data up front, storing it to disk and loading it again at training time. This process often involves manual steps and inhibits the possibility to make changes to which exact data are used. For example, using different thresholds to determine which regions to train on. Also, many copies of the same data corresponding to different hyperparameter settings of the data loader are stored.

We have developed a dataloader for BigWig files that addresses these issues by leveraging a novel GPU-based decompression library and the consistent input dimensionality common in standard machine learning applications.

## 2 Description

**Figure 1.**
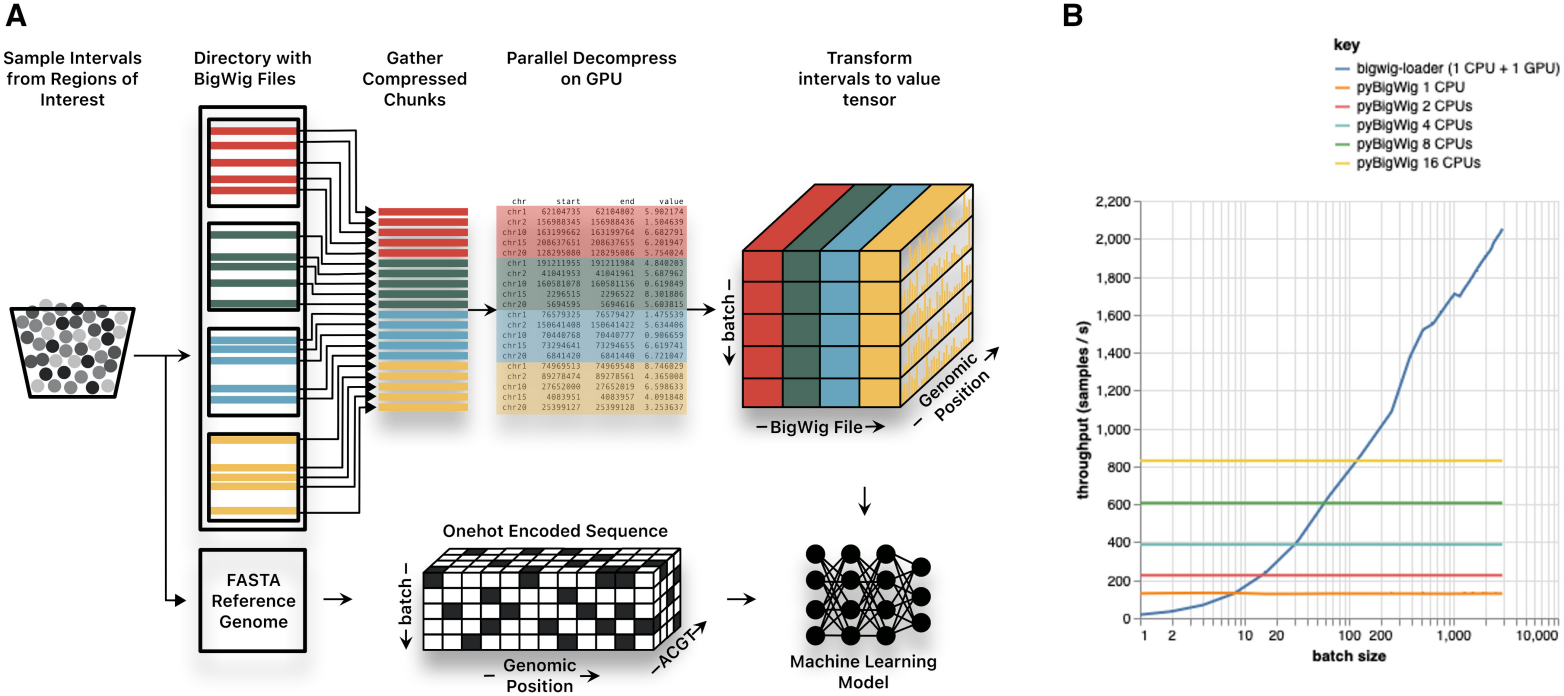
(A) Overview of the dataloading process in the bigwig-loader library. A batch of fixed length intervals are sampled from a general set of (larger) regions of interest. This can, for instance, be all regions used in either the train, validation, or test split. The library also contains functionality to create such regions of interest based on a value threshold. For the sampled intervals, relevant compressed chunks from all bigwig files are pulled from disk, decompressed and converted to a value tensor. Additionally genomic sequences are loaded and optionally one-hot encoded, so that now both input and target tensors are available for the typical supervised machine learning methods developed for this type of data. Machine learning models (right bottom) or code to train them are not part of this library. (B) Comparison between throughput of pyBigWig using multiple CPU’s and bigwig-loader. The number of samples pyBigWig can load per second is only dependent on the number of CPU cores used, not on the batch size. Also note that the relationship between the number of CPU’s and data throughput is not linear because multiprocessing has an overhead. When just a few samples are needed, pyBigWig is faster. When more than a few training examples are needed, as is the case for machine learning applications, bigwig-loader is the faster alternative.

When designing a data loader for machine learning, one can make certain assumptions that *pyBigWig*, as an all-purpose library, cannot make. For instance, one can assume that the sequence length corresponding to the epigenic profile will be the same in each training example and each training batch will have data from an equal number of BigWig files. This makes it possible to vectorize a lot the processing needed in the several steps to go from a set of BigWig files and batches of intervals to batches of profiles (See Fig. 1A).

To maximize data throughput, we take advantage of NVIDIA’s nvcomp library, which provides high-performance, GPU-accelerated compression and decompression API’s (https://developer.nvidia.com/nvcomp). More specifically, we use the nvcomp deflate decompressor to parallelize and speed up decompression of chunks of interval data across multiple BigWig files. In addition, we implemented a cuda kernel that subsequently transforms sets of intervals with values to value profiles using cupy (Okuta *et al.* 2017). Since cupy supports the cuda array interface and DLPack, zero-copy data exchange of the resulting tensor to deep learning frameworks like Pytorch and Tensorflow is possible (Abadi *et al.* 2015, Paszke *et al.* 2019).

When designing bigwig-loader, we aimed for data-loading as its singular, focused functionality in line with the Unix mentality of doing one thing and doing it well. This ensures that researchers with different preference for machine learning frameworks and higher-level training libraries can use it without being forced into a specific toolstack or workflow. The simplest use case is to query a set of BigWig files with a list of (same length) intervals and to receive back a value tensor of size *number of intervals x number of BigWig files x interval length*. Besides that, the library contains functions to randomly sample intervals from a set of genomic regions of interest. These would typically be train, validation, test regions. Also, functionality is included for defining these regions based on a value threshold, i.e. regions where at least one of the BigWig files exceeds that threshold. Lastly, the library contains functionality to load the corresponding sequences from the reference genome using the pyfaidx library (Shirley *et al.* 2015). Including the lookup of the sequence is also useful because the sampler can discard intervals containing a large amount of unknown bases and sample again, a trick also used in the sampler of Selene (Chen *et al.* 2019). Loading both the sequences and the epigenetic profiles provides the features and target tensors needed for machine learning methods typically trained on this datatype.

The library contains a set of unit tests, the most important one being the agreement with PyBigWig when tested on a set of BigWig files.

### 2.1 Example of typical use

The following code snippet showcases an example:

**import** pandas as pd

**from** bigwig_loader.dataset **import** BigWigDataset

train_regions = pd.read_csv(“train_regions.tsv”, sep=“\t”)

dataset = BigWigDataset(

   regions_of_interest=train_regions ,

   bigwig_path=/path/to/my/bigwig/directory ,

   reference_genome_path=/path/to/reference_genome.fasta,

   sequence_length = 1000,

   center_bin_to_predict = 1000,

   batch_size = 256,

   super_batch_size = 1024,

   batches_per_epoch = 20,

   maximum_unknown_bases_fraction = 0.1,

   sequence_encoder=”onehot”,

)

*# use in training loop. Model is not part of the library*

for encoded_sequence, epigenetic_profiles in dataset:

   model.train_step (

   features=encoded_sequence ,

   target=epigenetic_profiles

)

### 2.2 Performance

In a data loading comparison with *PyBigWig*, we find that *bigwig-loader’s* GPU decompression of BigWig chunks and GPU post-processing of interval data significantly enhances data throughput for larger batch sizes. While PyBigWig excels when loading a small number of training examples, *bigwig-loader* outperforms it when more than a few examples are needed, as is typical in machine learning applications. When using a single CPU for *pyBigWig* (and one CPU plus one GPU for *bigwig-loader*), the break-even point lays around eight samples (Table 1). To emulate a more realistic scenario where multiple CPU’s are used alongside *PyBigWig*, we created a PyTorch dataloader with multiple workers (Fig. 1B). In this setup, using 16 CPU’s for *PyBigWig* shifts the break-even point, where empbigwig-loader becomes faster, to a batch size of approximately 128 samples.

**Table 1.** Synthetic data loading benchmark of loading batches of intervals from a set of 113 BigWig files.

Batch size	bigwig-loader (s)	PyBigWig (s)	Time difference (%)
1	0.057	0.007	814.3
8	0.0615	0.060	102.5
64	0.099	0.496	−490.9
256	0.235	1.98	−842.6
1024	0.599	7.970	−1330.6
2048	1.074	15.897	−1480.2

Benchmarks were all run on the same machine at which bigwig-loader used a NVIDIA A100 GPU.

It’s worth noting that the batch size set for the data loader can be significantly larger than the batch size used for each stochastic gradient descent step, a crucial hyperparameter of the optimizer. This is because the batches being processed at once by the dataloader can be trivially split into multiple smaller batches if that improves the training process.

## 3 Conclusion

With *bigwig-loader*, we provide a dataloader for epigenetic profiles from BigWig files specifically geared toward machine learning. Not only using the GPU for the training of the model, but also for the preprocessing of the data, makes a simpler and more streamlined process for learning possible. We hope that because of this new library it becomes easier to run experiments that otherwise would have made it necessary to reprocess and persist training data, thereby indirectly improving the quality of the models.
